# Elimination of Carryover Contamination in Real-Time Reverse Transcriptase Loop-Mediated Isothermal Amplification for Rapid Detection of the SARS-CoV-2 Virus in Point-of-Care Testing

**DOI:** 10.3389/fcimb.2022.856553

**Published:** 2022-04-20

**Authors:** Than Linh Quyen, Aaydha Chidambara Vinayaka, Mohsen Golabi, Huynh Van Ngoc, Dang Duong Bang, Anders Wolff

**Affiliations:** ^1^Biolabchip Group, Department of Bioengineering, Technical University of Denmark, Lyngby, Denmark; ^2^Laboratory of Applied Micro and Nanotechnology (LAMINATE), Department of Bioengineering, Technical University of Denmark, Lyngby, Denmark

**Keywords:** carryover contamination, loop-mediated isothermal amplification (LAMP), SARS-CoV-2, COVID-19, Cod uracil DNA glycosylase (Cod-UNG), on-site testing, point-of-care

## Abstract

Loop-mediated isothermal amplification (LAMP) is being used as a robust rapid diagnostic tool to prevent the transmission of infectious diseases. However, carryover contamination of LAMP-amplified products originating from previous tests has been a problem in LAMP-based bio-analytical assays. In this study, we developed a Cod-uracil-DNA-glycosylase real-time reverse transcriptase LAMP assay (Cod-UNG-rRT-LAMP) for the elimination of carryover contamination and the rapid detection of SARS-CoV-2 in point-of-care (POC) testing. Using the Cod-UNG-rRT-LAMP assay, the SARS-CoV-2 virus could be detected as low as 2 copies/µl (8 copies/reaction) within 45 min of amplification and 2.63 ± 0.17 pg (equivalent to 2.296 × 10^9^ copies) of contaminants per reaction could be eliminated. Analysis of clinical SARS-CoV-2 samples using the Cod-UNG-rRT-LAMP assay showed an excellent agreement with a relative accuracy of 98.2%, sensitivity of 97.1%, and specificity of 95.2% in comparison to rRT-PCR. The results obtained in this study clearly demonstrate the feasibility of the use of the Cod-UNG-rRT-LAMP assay for applications toward the POC diagnosis of SARS-CoV-2 and on-site testing of other pathogens.

## Introduction

COVID-19 is an ongoing pandemic caused by a new severe acute respiratory syndrome coronavirus 2 (SARS-CoV-2) (https://www.who.int/emergencies/diseases/novel-coronavirus-2019/technical-guidance/naming-the-coronavirus-disease-(covid-2019)-and-the-virus-that-causes-it). The virus spreads very easily through contact, droplets, airborne, fomite, fecal–oral, blood-borne, mother-to-child, and animal-to-human (https://www.who.int/news-room/commentaries/detail/transmission-of-sars-cov-2-implications-for-infection-prevention-precautions). The disease was identified for the first time in December 2019 in Wuhan, China (Zhu et al., 2020), and quickly became a Public Health Emergency of International Concern on January 30, 2020 (https://www.who.int/publications/m/item/covid-19-public-health-emergency-of-international-concern-(pheic)-global-research-and-innovation-forum). On March 11, 2020, the World Health Organization (WHO) declared the COVID-19 pandemic with more than 118,000 infections and 4,291 deaths in 114 countries (https://www.who.int/director-general/speeches/detail/who-director-general-s-opening-remarks-at-the-media-briefing-on-covid-19—11-march-2020). As of January 27, 2022, COVID-19 has affected 220 countries and territories with more than 434 million confirmed cases and more than 5.9 million deaths (https://covid19.who.int/). Besides the health effects, the pandemic has caused social turmoil and economic disruption (https://www.ilo.org/wcmsp5/groups/public/—ed_dialogue/—act_emp/documents/publication/wcms_745024.pdf; https://www.un.org/development/desa/dspd/; https://www.worldbank.org/en/news/factsheet/2020/07/13/economic-and-social-impacts-of-covid-19-update-from-listening-to-tajikistan; https://en.unesco.org/sites/default/files/issue_1_en_culture_covid-19_tracker.pdf; https://en.unesco.org/covid19/educationresponse). Therefore, preventing the transmission of the virus will reduce remarkably the negative effects on the health, economy, and society.

Currently, reverse transcript real-time polymerase chain reaction (rRT-PCR) is being used widely as a standard method in the laboratories to detect the presence of SARS-CoV-2 in the clinical samples (https://www.who.int/docs/default-source/coronaviruse/protocol-v2-1.pdf; Corman et al., 2020). However, rRT-PCR is a time-consuming assay that requires sophisticated laboratory facilities and well-trained personnel and may exhibit high inhibitory effects. In recent years, loop-mediated isothermal amplification (LAMP) has been demonstrated as a powerful alternative to overcoming the drawbacks of PCR in clinical diagnostics (Rahman et al., 2017; Kaur et al., 2018; Rabe and Cepko, 2020; Lalli et al., 2021). LAMP, being an isothermal amplification technique, can be performed at a constant temperature between 60°C and 65°C using a simple heat block for amplification. LAMP has several advantages such as fast amplification, higher sensitivity, higher specificity, and more resistance to inhibitors from clinical samples than PCR (Notomi, 2000; Nagamine et al., 2002; Francois et al., 2011; Njiru, 2012; Gotoh et al., 2013; Stedtfeld et al., 2014; Kosti et al., 2015; Velders et al., 2018; Lalli et al., 2021). In addition, the LAMP reaction produces a huge amount of amplified products that are easily detectable by the naked eyes based on color change (Almasi et al., 2013; Xie et al., 2014; Tanner et al., 2015; Dao Thi et al., 2020). Besides the amplified product, the LAMP reaction also produces a large amount of the by-product (magnesium pyrophosphate) that allows visualization of results using a turbidimeter (Mori et al., 2004) or even by naked eye (Yang et al., 2010). With these advantages, LAMP may be considered as an alternative to PCR in rapid POC diagnostic applications (Sun et al., 2015; Nguyen et al., 2020).

However, carryover contamination has been a big problem in LAMP assays (Borst et al., 2004; Tomita et al., 2008; Bao et al., 2020). Aerosols with high concentration of amplified products may easily be formed while handling reaction tubes, if not done carefully, leading to contamination of the surrounding area. As a consequence, carryover contamination can take place through contaminated pipets, reagents, gloves, work surface, or clothes (Kwok and Higuchi, 1989; Kitchin et al., 1990) and might result in false-positive results in LAMP assays. Researchers have developed several methods to control the carryover contamination in LAMP assay. Kil et al., (2015) used uracil-DNA-glycosylase (UNG) to destroy carryover amplified products in LAMP reaction (Kil et al., 2015; Tang et al., 2016). This method requires the incorporation of deoxyuridine triphosphate (dUTP) into the amplified product by a DNA polymerase during amplification and excision of those uracils in the amplified product by UNG. Ma et al. (2017) reported another method to control carryover contamination by designing recognition sites for restriction endonuclease *Gsu* I in primers (Ma et al., 2017). In this approach, restriction enzyme *Gsu* I recognizes restriction sites and breaks the products that contained recognition sites. However, this approach requires high temperature to deactivate the restriction enzyme that may adversely affect LAMP efficiency. Moreover, the addition of restriction sites in LAMP primers also increases the complexity of the LAMP primer design. Recently, Bao et al. (2020) reported a novel CUT-LAMP method, which was based on the CRISPR/Cas9 cleavage, to eliminate the carryover contamination issue (Bao et al., 2020). In the CUT-LAMP approach, the FIP primer was modified by an addition of CC bases to the linker between F2 and F1c regions and generated GG bases in LAMP amplified products. An NGG (N stands for any other base), called PAM (protospacer adjacent motif) site, is cleaved by the Cas9/sgRNA system. The CUT-LAMP could be performed at room temperature and require no inactivation step. However, the method has a lower efficiency of the elimination of carryover contaminants than UNG (https://webshop.tataa.com/dokument/ArcticZymes_CodUNG_Flyer.pdf). The use of UNG cleavage is, therefore, considered as an appropriate approach to control the carryover contamination of LAMP products so far.

In this study, we developed Cod-UNG-rRT-LAMP for the simultaneous rapid detection and elimination of carryover contamination of SARS-CoV-2 LAMP products in a simple preparation step. We investigated the sensitivity of the assay and further evaluated the assay with 55 clinical samples. Furthermore, we tested the Cod-UNG-rRT-LAMP reaction with different detection methods to detect SARS-CoV-2 for future applications toward POC diagnostics.

## Materials and Methods

### LAMP Primers and Cod-UNG-rRT-LAMP Reaction

A LAMP primer set (**Table 1**) targeting gene N (Nucleocapsid phosphoprotein) of SARS-CoV-2 located at the region between nucleotides 28,501–28,709 of the genome (LC547533.1) was used for this study (Zhang et al., 2020). The specificity of the primer set is shown in **Figure S1**. The real-time reverse transcriptase LAMP (rRT-LAMP) assay was carried out in 20 μl of master mixture containing 0.2 μM of F3, 0.2 μM of B3, 1.6 μM of FIP, 1.6 μM of BIP, 0.8 μM of LF, 0.8 μM of LB (Integrated DNA Technologies, Leuven, Belgium), 0.35 mM of dATP, 0.35 mM dGTP, 0.35 mM of dCTP, various concentrations of dTTP and dUTP ranging from 0 to 0.35 mM (Thermo Fisher Scientific, Roskilde, Denmark), 0.25 M betaine (Sigma-Aldrich, Denmark), 6 U of Warmstart^®^ RTx Reverse Transcriptase, 8 U of *Bst* Warmstart^®^ 2.0 DNA polymerase, 1× isothermal amplification buffer (New England Biolabs), 0.01 U of Cod-UNG, 2 µM of SYTO 9, sterilized water, and RNA template.

**Table 1:** LAMP primer sets used in this study (Zhang et al., 2020).

Name	Sequences (5′–3′)	GC content (%)
F3	ACCGAAGAGCTACCAGACG	57.9
B3	TGCAGCATTGTTAGCAGGAT	45
FIP	TCTGGCCCAGTTCCTAGGTAGTTCGTGGTGGTGACGGTAA	55
BIP	AGACGGCATCATATGGGTTGCACGGGTGCCAATGTGATCT	52.5
LF	CCATCTTGGACTGAGATCTTTCATT	40
LB	ACTGAGGGAGCCTTGAATACA	47.6

The Cod-UNG-rRT-LAMP reactions were incubated at 25°C for 5 min, then performed on an Mx3005P system (Stratagene, AH Diagnostics, Denmark) at 55°C for 5 min followed by 65°C for 60 min, and terminated by heating to 90°C for 5 min. The fluorescence signal was recorded every minute of amplification.

### Real-Time Reverse Transcriptase PCR

Real-time reverse transcriptase PCR (rRT-PCR) targeting the E-gene of SARS-CoV-2 was used as the reference method as described previously (Corman et al., 2020; Jørgensen et al., 2021) to evaluate the performance of Cod-UNG-rRT-LAMP. In short, each 20 µl reaction contains 400 nM E_Sarbeco_forward (F), 700 nM E_Sarbeco_reverse (R) primers, 150 nM E_Sarbeco_P1 probe, and TaqMan Fast Virus 1-Step Master Mix (FV1S MM, Thermo Fisher Scientific) supplemented with 0.2 mM dUTP and 8 µl of the target sample. rRT-PCR was performed in a LightCycler 480 system with the following conditions: 55°C for 20 min, 95°C for 5 min, followed by 50 cycles of 95°C for 15 s, 60°C for 60 s, and 72°C for 30 s.

### Simulating Carryover Contamination in Cod-UNG-rRT-LAMP Reaction

An amplified LAMP product incorporated with dUTP from the previous reaction was used for simulating carryover contamination in the Cod-UNG-rRT-LAMP reaction. An amplified product from an rRT-LAMP reaction performed only with dTTP was used as control. A serial 10-fold dilution of the products was prepared and used as a template in the Cod-UNG-rRT-LAMP reaction.

### Analytical Precision

The sensitivity of the Cod-UNG-rRT-LAMP reaction was evaluated using a clinical SARS-CoV-2-positive sample collected at Hvidovre Hospital, Denmark. The RNA sample was extracted and purified by MagNA Pure 96 DNA and Viral NA Small Volume Kit (Life Science, Roche, Denmark) following the manufacturer’s instruction. A serial 5-fold dilution of the extracted RNA was prepared in phosphate-buffered saline (PBS), and 4 µl of each dilution was used as the template in the Cod-UNG-rRT-LAMP reaction.

The developed method was used to test 55 throat swab samples (clinical SARS-CoV-2 samples) collected in Denmark, during the COVID-19 pandemic, which included 20 negative and 35 positive samples. These clinical samples were confirmed by rRT-PCR as mentioned above. Out of the 35 positive samples, 17 samples had C_t_ in the range 9–15, 12 samples had C_t_ in the range 15–20, and 6 samples had C_t_ in the range 20–26 (**Table S1**). The RNA was extracted from these samples as described above. Four microliters of the extracted RNA was used as the template in the Cod-UNG-rRT-LAMP reaction. In addition, the developed method was also tested using culture of SARS-CoV-2 spiked-in negative throat swab samples processed *via* a simple boiling method wherein the samples were heated at 95°C for 5 min.

The precision of the Cod-UNG-rRT-LAMP assay was evaluated by comparing with PCR. The precision of the method was evaluated based on relative accuracy, relative, specificity, relative sensitivity, and Cohen’s kappa index as described previously (Quyen et al., 2019b) (see **Supplementary Data** for details).

### Detection of the Cod-UNG-rRT-LAMP Product

The products of the Cod-UNG-rRT-LAMP reaction were analyzed with four different detection methods: a) real-time fluorescence detection using DNA-intercalating dye; b) real-time turbidity detection; c) end-point direct visual detection by the naked eye, and d) gel electrophoresis.

- Real-time fluorescence detection method: 5 mM SYTO-9 (Invitrogen, Carlsbad, CA, USA) was diluted in sterilized water and used at a final concentration of 2 µM in the Cod-UNG-rRT-LAMP assay. The dye was added into the master mixture before amplification (Quyen et al., 2019a), and the fluorescence signal was measured by a real-time PCR system (Mx3005P) as mentioned above.
- Real-time turbidity detection method: 100 mM MgSO_4_ (New England Biolabs, Ipswich, MA, USA) was added to the reaction mixture at a final concentration of 1.5 mM in the Cod-UNG-rRT-LAMP reaction. The real-time turbidity was measured by an *in-house* developed point-of-care device—the PATHPOD system (PATHPOD, European patent application no. 20173505.7-EPO, www.vivaldi-ia.eu; www.coronadx-project.eu/diagnostic-kits/pathpod/).
- End-point direct visual detection method by the naked eye: for the end-point detection of amplified products, 5 mM SYTO-24 (Invitrogen, USA) was diluted in sterilized water and added directly to the Cod-UNG-rRT-LAMP-amplified products at a final concentration of 100 µM. The change in the color of the reaction products was monitored by the naked eye.
- Gel electrophoresis detection: 8 μl of amplified LAMP products was loaded on 2% agarose gel containing 1× of SYBR^®^ Safe DNA Gel Stain (Invitrogen, USA). The gel electrophoresis was carried out at 110 V for 60 min and observed under a Bio-Rad Gel Doc 2000 UV transilluminator (Bio-Rad Life Science, Denmark).

## Results and Discussions

### Effect of dUTP Concentration on the rRT-LAMP Reaction

**Figure 1:**
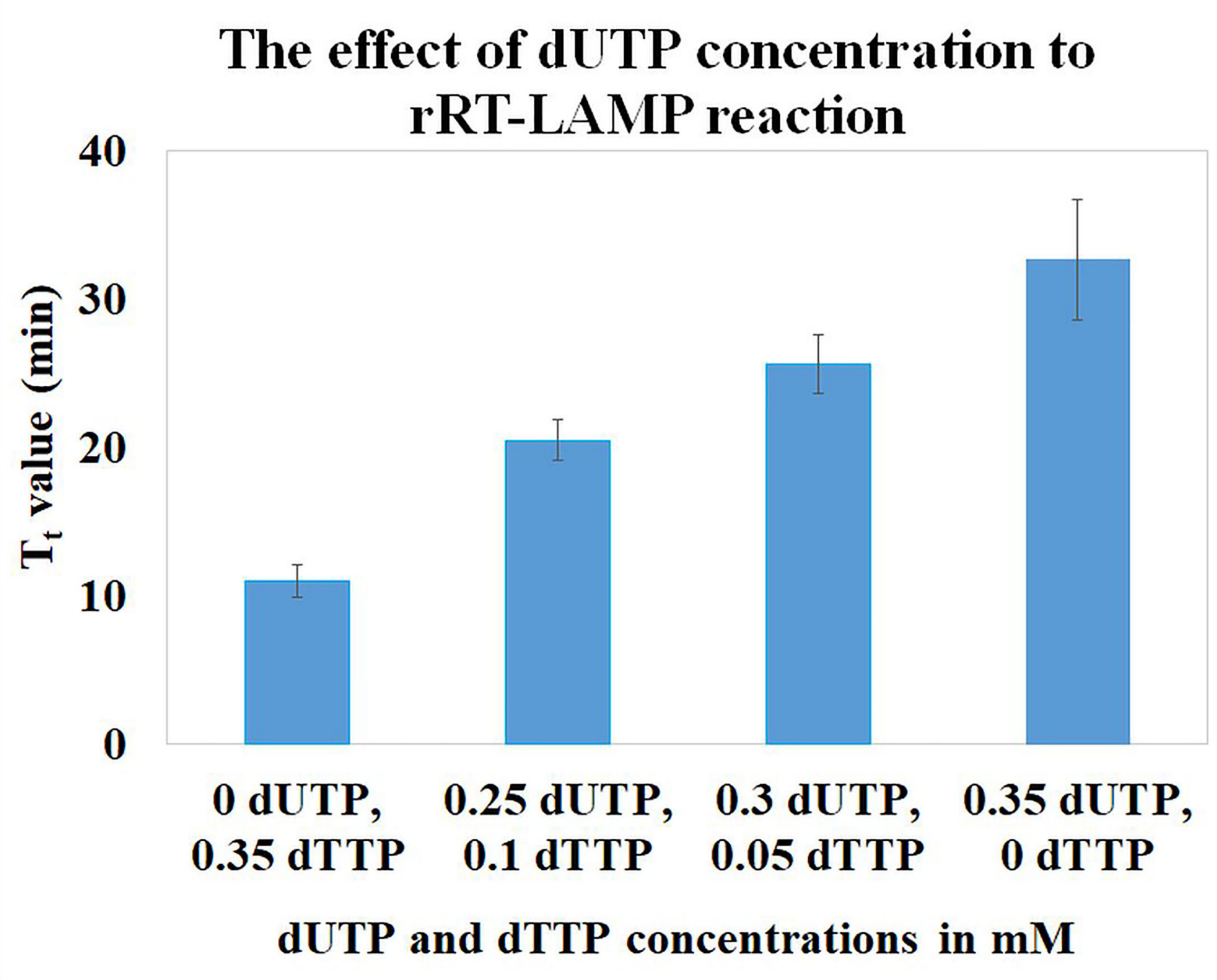
The effect of dUTP concentrations on rRT-LAMP reaction.

To investigate the effect of dUTP concentration on the LAMP assay, mixtures of different concentrations of dUTP and dTTP were tested while concentrations of dATP, dCTP, and dGTP were unchanged in the LAMP reactions. In general, the LAMP reactions are performed with 0.35 mM concentration of each dNTP. In this study, different concentrations of dUTP and dTTP (0 and 0.35, 0.25 and 0.1, 0.3 and 0.05, and 0.35 and 0 mM, respectively) were studied, while maintaining the total concentration of dUTP plus dTTP at 0.35 mM. We observed an increase in T_t_ (threshold time) values in the LAMP reactions when increasing the concentration of dUTP. However, in order to maintain the total concentration of dUTP and dTTP in the reaction mixture constant at 0.35 mM, the concentration of dTTP is simultaneously reducing (**Figure 1**). In the reaction containing 0 mM dUTP and 0.35 mM dTTP, the T_t_ value was 11.0 ± 1.0 min. While in the reactions containing 0.25 and 0.1, and 0.3 and 0.05 mM of dUTP and dTTP, the T_t_ values were increased to 20.5 ± 1.4 and 25.6 ± 1.9 min, respectively. In the reaction containing 0.35 mM dUTP and no dTTP, the T_t_ value was 32.7 ± 4.1 min. The results indicated that dUTP had a partial inhibitory effect on the rRT-LAMP reaction.

The efficiency of the elimination of carryover contaminants depends on the incorporation of dUTP in LAMP-amplified products by DNA polymerase. In this study, *Bst* 2.0 Warmstart^®^ DNA Polymerase was used since this polymerase exhibits several advantages such as higher amplification speed, yield, salt tolerance, and high efficiency incorporation of dUTP in the LAMP reactions (https://international.neb.com/products/m0538-bst-20-warmstart-dna-polymerase#Product%20Information). In general, it is recommended to use 50% dUTP and 50% dTTP in the reaction mixtures in order to obtain the highest-efficiency incorporation of dUTP without significant inhibition of the reaction. However, at this ratio, the efficiency of the elimination of carryover contaminants may not be significant since the polymerase prefers to use dTTP than dUTP. Although there was a delay in the amplification when a higher dUTP concentration was used, the elimination of the carryover contaminant would be expected to be better than the use of 50% dUTP and 50% dTTP. Kil et al. (2015) studied the effect of the dUTP concentration on the LAMP reaction, but they could not observe any inhibitory effect since their reaction was observed at the end point after 60 min.

### Elimination of Carryover Contamination by the Cod-UNG-rRT-LAMP Reaction

The elimination of carryover contamination also depends on the hydrolysis of uracils incorporated in the contaminants by UNG. Among different UNGs, Cod-UNG was selected for this study because this enzyme was active at a wider range of temperatures ranging from 20°C to 40°C, and the activity was lost at above 42°C. This is a great advantage since, using Cod-UNG in combination with *Bst* 2.0 Warmstart^®^ DNA Polymerase, the experiments can be performed at room temperature as the *Bst* 2.0 Warmstart gets activated at a temperature above 45°C. As a result, the experimental setup is simpler and faster.

**Figure 2:**
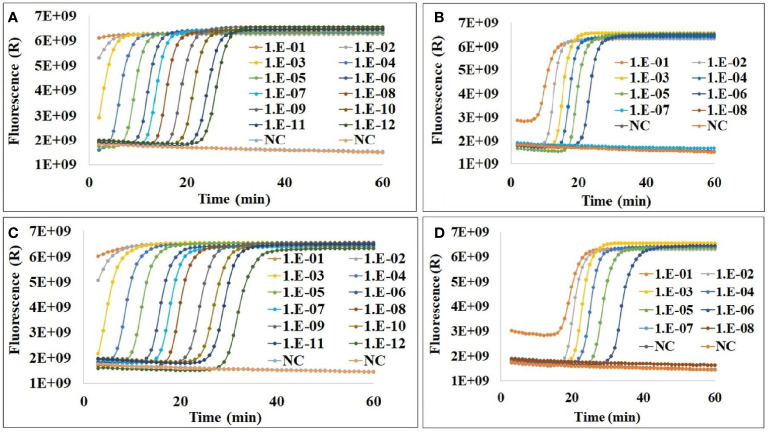
The elimination of carryover contamination in Cod-UNG-rRT-LAMP reaction **(A)** containing 0.3 mM dUTP and 0.05 mM dTTP and using log_10_ dilution of an amplified product from an rRT-LAMP reaction (containing only dTTP) as control template; **(B)** containing 0.3 mM dUTP and 0.05 mM dTTP and using log_10_ dilution of an amplified product from a Cod-UNG-rRT-LAMP reaction (containing 0.3 mM dUTP and 0.05 mM dTTP) as template; **(C)** containing 0.35 mM dUTP and 0 mM dTTP and using log_10_ dilution of the amplified product from the rRT-LAMP reaction (containing only dTTP) as control template; and **(D)** containing 0.35 mM dUTP and 0 mM dTTP and using log_10_ dilution of the amplified product from the Cod-UNG-rRT-LAMP reaction (containing 0.35 mM dUTP and 0 mM dTTP) as template.

To evaluate the effect of elimination of carryover contamination of Cod-UNG in the rRT-LAMP reaction, two concentrations of dUTP and dTTP such as 0.3 and 0.05 and 0.35 and 0 mM were selected and tested. The LAMP-amplified products of the two reactions (containing 0.3 and 0.05, and 0.35 and 0 mM dUTP and dTTP) were compared with an amplified LAMP product performed in the absence of dUTP as a control. The Cod-UNG-rRT-LAMP assays (performed under both 0.3 and 0.05 and 0.35 and 0 mM dUTP and dTTP conditions) were positive only with the LAMP-amplified templates containing dTTP in the dilutions ranging from 1.E-1 to 1.E-12 within 40 min (**Figures 2A, C**). However, when using templates containing 0.3 and 0.05 mM dUTP and dTTP in the reactions, Cod-UNG-rRT-LAMP could only eliminate contaminants from 1.E-7 dilution onward (**Figure 2B**). Similar results were also observed when using templates incorporated with 0.35 mM dUTP in the Cod-UNG-rRT-LAMP reaction (**Figure 2D**). After LAMP amplification, 13.17 µg of amplified products can be generated. This infers that the Cod-UNG-rRT-LAMP reaction could eliminate ~2–3 pg (2.63 ± 0.17 pg, equivalent to 2.296 × 10^9^ copies) of undesirable contaminant products in the reaction. The elimination of contaminant DNA observed using Cod-UNG in this study is better than that using UNG (10^-4^ pg/reaction) (Tang et al., 2016) and similar to CUT-LAMP (Bao et al., 2020), reported previously. The efficiency of elimination of the carryover contamination of Cod-UNG-rRT-LAMP depends on (1) the incorporation of dUTP in the LAMP product that was responsible by *Bst* 2.0 Warmstart^®^ DNA polymerase and (2) the hydrolysis of uracils incorporated in the contaminants that was responsible by Cod-UNG. Therefore, we believe that different primer sequences or different targets will not affect to the efficiency of the Cod-UNG-rRT-LAMP reaction.

### Sensitivity of the Cod-UNG-rRT-LAMP Reaction for Detection of SARS-CoV-2

**Figure 3:**
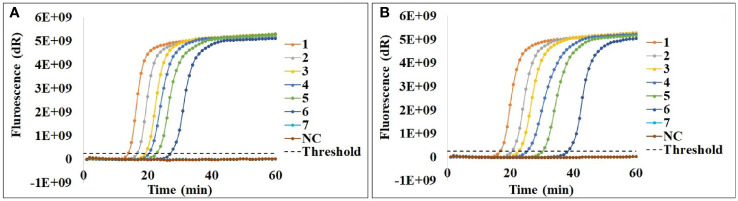
Sensitivity of the Cod-UNG-rRT-LAMP assay containing **(A)** 0.3 mM dUTP and 0.05 mM dTTP and **(B)** 0.35 mM dUTP and no dTTP. In both **(A)** and **(B)**, a serial 5-fold dilution of a SARS-CoV-2 positive clinical sample was prepared. 1:5 times dilution, 2:25 times dilution, 3:125 times dilution, 4:625 times dilution, 5:3,125 times dilution, 6:15,625 times dilution, 7:78,125 times dilution, NC, negative control.

The sensitivity of the Cod-UNG-rRT-LAMP assay was investigated with two different combinations of dUTP and dTTP concentrations of 0.3 and 0.05 and 0.35 and 0 mM using a serial 5-fold dilution of the RNA sample with an original concentration of ˜ 29,176 viral RNA copies/μl. A sensitivity of ˜ 2 copies/µl or 8 copies/reaction (15.62 times dilution) within 27 min (**Figure 3A**) was observed when using the Cod-UNG-rRT-LAMP reaction containing 0.3 and 0.05 mM dUTP and dTTP. Similar LOD was achieved for the Cod-UNG-rRT-LAMP reaction with 0.35 and 0 mM dUTP and dTTP. However, the T_t_ of the reaction (containing 0.35 and 0 mM dUTP and dTTP) was delayed by 11 min (T_t_ at 38 min) compared to the reaction containing 0.3 and 0.05 mM dUTP and dTTP (T_t_ at 27 min) (**Figure 3B**). The delay of the T_t_ value in the Cod-UNG-rRT-LAMP containing 0.35 mM dUTP and 0 mM dTTP may be due to the effect of the incorporation of dUTP to amplified products by the *Bst* polymerase. The elimination efficiency of contaminant of the Cod-UNG-rRT-LAMP assay was similar in both 0.3 and 0.05 and 0.35 and 0 mM dUTP and dTTP concentrations, but the Cod-UNG-rRT-LAMP reaction containing 0.3 and 0.05 mM dUTP and dTTP was faster. It was therefore selected for further study.

### Detection of SARS-CoV-2 by the Cod-UDG-rRT-LAMP Using Clinical Samples

The efficiency of the Cod-UDG-rRT-LAMP assay containing 0.3 and 0.05 mM dUTP and dTTP was further evaluated using clinical samples and adapted for SARS-CoV-2 rapid diagnostics. A total of 55 clinical SARS-CoV-2 samples which included 35 positive (with C_t_ ranging from 9 to 26 as mentioned above) and 20 negative RNA samples were collected and tested by the Cod-UDG-rRT-LAMP assay in parallel with PCR as control methods. The results showed that of 55 samples, 34 samples were positive and 21 samples were negative in the developed method (**Table 2** and **Table S1**). In comparison to rRT-PCR, Cod-UDG-rRT-LAMP showed 98.2% accuracy, 95.2% specificity, and 97.1% sensitivity. Cohen’s kappa index also showed an excellent agreement (0.97) between Cod-UDG-rRT-LAMP and rRT-PCR (**Table 2**).

**Table 2:** Comparison of the Cod-UDG-rRT-LAMP assay to rRT-PCR for detection of SARS-CoV-2 in 55 clinical samples.

Samples	Cod-UNG rRT-LAMP	rRT-PCR
Positive	34	35
Negative	21	20
Total	55	55
**Comparison of Cod-UNG rRT-LAMP and rRT-PCR**		
Relative accuracy (AC%) Relative specificity (SP%) Relative sensitivity (SE%) Cohen’s kappa index		98.2
		95.2
		97.1
		0.97

### Effect of the Cod-UNG-rRT-LAMP Reaction on Different Detection Principles

**Figure 4:**
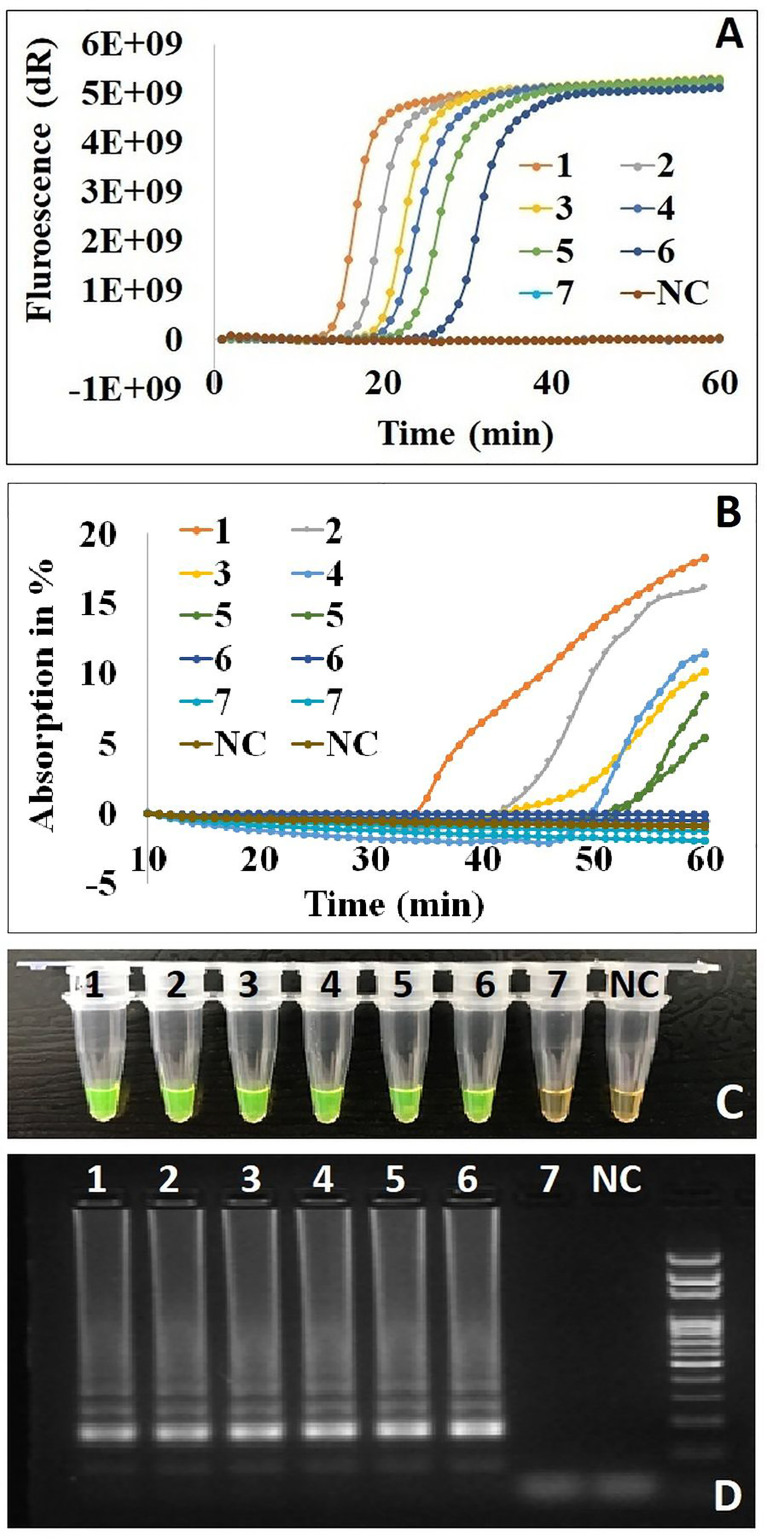
The effect of Cod-UNG-rRT-LAMP assay in different detection principles. **(A)** Fluorescence detection method, **(B)** turbidity detection method, **(C)** direct visual detection method (in positive reaction color changed from orange to green, in negative reaction the color remains orange), and **(D)** gel electrophoresis method. In **(A–D)**, a serial 5-fold dilution of a positive clinical sample was prepared. 1:5 times dilution, 2:25 times dilution, 3:125 times dilution, 4:625 times dilution, 5:3,125 times dilution, 6:15,625 times dilution, 7:78,125 times dilution, NC, negative control.

The Cod-UNG-rRT-LAMP reaction was investigated with different detection principles: real-time fluorescence detection, real-time turbidity detection, direct visual detection by the naked eyes, and gel electrophoresis detection. For the real-time fluorescence detection principle, an LOD of ˜ 2 copies/µl (or 8 copies/reaction) was achieved by the Cod-UNG-rRT-LAMP assay and was comparable to rRT-LAMP (**Figure 4A** and **Figure S2A**). A similar LOD (˜ 2 copies/µl) was observed in the direct visual detection approach or gel electrophoresis detection (**Figures 4C, D**), while for real-time turbidity detection, an LOD of ˜ 10 copies/µl (60 copies/reaction) was observed for both Cod-UNG-rRT-LAMP and rRT-LAMP. This LOD was 5 times higher compared to the real-time fluorescence detection principle (**Figure 4B** and **Figure S2B**). The higher LOD was probably due to the addition of MgSO_4_ for turbidity generation in the reaction, which partly inhibited the LAMP amplification in our experience (**Figure 4B** and **Figure S2B**). These results inferred that Cod-UNG had no effect on the Cod-UNG-rRT-LAMP reaction and could be adaptable to various detection principles in POC testing.

### Effect of Boiling Method (Sample Preparation) on the Cod-UNG-rRT-LAMP Assay

**Figure 5:**
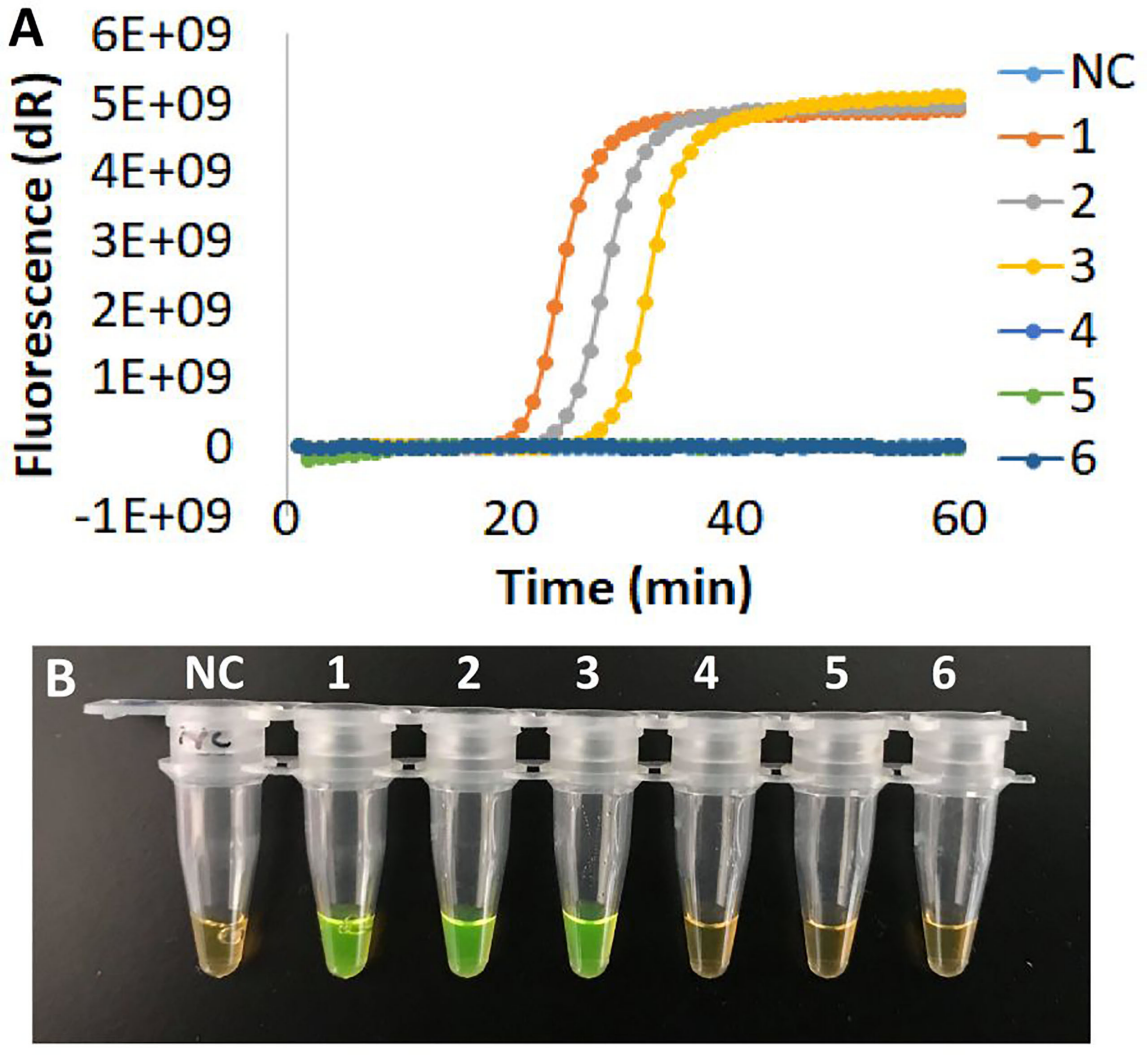
The effect of Cod-UNG-rRT-LAMP assay using boiling samples. **(A)** Fluorescence detection method, **(B)** direct visual detection method. In both **(A)** and **(B)**, a serial 10-fold dilution of a culture virus in negative throat swabs sample was prepared by boiling method at 95°C for 5 min. NC, negative control, 1: 10^2^ times dilution, 2: 10^3^ times dilution, 3: 10^4^ times dilution, 4: 10^5^ times dilution, 5: 10^6^ times dilution, 6: 10^7^ times dilution.

We further investigated the use of the Cod-UNG-rRT-LAMP assay for the detection of SARS-CoV-2 in the throat swab samples processed by the simple boiling method. For this investigation, a serial 10-fold dilution of culture of a SARS-CoV-2 viral sample was prepared in negative throat swab matrix (prepared by mixing 1 swab in 300 µl of PBS) and heated at 95°C for 5 min. The reaction was positive up to 10^4^ dilutions (corresponding to 1,390 copies/reaction), and the samples remained undetectable at higher dilutions (**Figure 5A**). Similar results were observed in the end-point direct visual detection method (**Figure 5B**) (Vinayaka et al., 2022).

Sample preparation has been a challenge in the POC quick diagnostic test. Sample preparation with the boiling method (heat lysis of cells at 95°C for 5 min) could be a simple approach that requires only a heat block or water bath. The method was previously reported with swab samples for the detection of SARS-CoV-2 (Dao Thi et al., 2020). As a result, it could reduce the analysis time and make the assay field applicable for preliminary quick testing at low-resource settings.

## Conclusion

We have developed the Cod-UNG-rRT-LAMP assay to overcome the carryover contamination problems in LAMP-based POC diagnostics and demonstrated it with SARS-CoV-2 diagnostics. The developed Cod-UNG-rRT-LAMP assay was sensitive and could detect SARS-CoV-2 down to ˜ 2 copies/µl (8 copies/reaction) within 45 min by the real-time fluorescence detection method or end-point direct visual detection method, or ˜ 10 copies/µl (60 copies/reaction) within 60 min by the real-time turbidity detection method using an in-house POC devise. The reaction can eliminate 2–3 pg of contaminants in the reaction. The results of the Cod-UNG-rRT-LAMP assay were comparable with those with PCR as the assay had a comparable analytical precision and clinical sensitivity, as well as accuracy. The results obtained in this study showed the greater potential of the Cod-UNG-rRT-LAMP assay for applications toward POC diagnosis.

## Data Availability Statement

The original contributions presented in the study are included in the article/**Supplementary Material**. Further inquiries can be directed to the corresponding author.

## Author Contributions

AW, DB, TQ, AV, MG, and HN designed the work. TQ performed the experiments. TQ, AW, DB, AV, MG, and HN wrote the manuscript. All authors contributed to the article and approved the submitted version.

## Funding

This study was supported by the Department of Biotechnology and Biomedicine, Technical University of Denmark, Denmark; EU H2020-funded projects CORONADX (grant no. 101003562, https://coronadx-project.eu/); VIVALDI (grant no. 773422, www.vivaldi-ia.eu); and the Danish government funding, COVIDTESTS.

## Conflict of Interest

The authors declare that the research was conducted in the absence of any commercial or financial relationships that could be construed as a potential conflict of interest.

## Publisher’s Note

All claims expressed in this article are solely those of the authors and do not necessarily represent those of their affiliated organizations, or those of the publisher, the editors and the reviewers. Any product that may be evaluated in this article, or claim that may be made by its manufacturer, is not guaranteed or endorsed by the publisher.
