# Supplementary material for: Elimination of Carryover Contamination in Real-Time Reverse Transcriptase Loop-Mediated Isothermal Amplification for Rapid Detection of the SARS-CoV-2 Virus in Point-of-Care Testing
Source: Front Cell Infect Microbiol. 2022 Apr 20;12:856553. doi: 10.3389/fcimb.2022.856553 (PMC9065284; doi:10.3389/fcimb.2022.856553)
Supplement: Supplementary file 1 [file DataSheet_1.pdf]

Supplementary Material

Evaluation of assays precision between real-time PCR and LAMP was calculated based on relative accuracy, relative specificity, relative sensitivity and Cohen's kappa index as described previously (Quyen at al., 2019b) using following formulas:

$$Relative\ accuracy\ AC(\%) = \frac{(\mathrm{PA} + \mathrm{NA})}{\mathrm{N}} \times 100$$

$$Relative\ specificity\ SP(\%) = \frac{\mathrm{NA}}{\mathrm{N}-} \times 100$$

$$Relative\ sensitivity\ SE(\%) = \frac{\mathrm{PA}}{\mathrm{N}+} \times 100$$

$$Cohen's\ Kappa\ index = \frac{\mathrm{P(o)} - \mathrm{P(e)}}{1 - \mathrm{P(e)}}$$

Where:

PA: the positive agreement between the real-time PCR and LAMP methods;
NA: the negative agreement between the real-time PCR and LAMP methods;
N: total number of samples (NA+PA+PD+ND);
PD: false positives in the LAMP method;
ND: false negatives in the LAMP method;
N-: total number of negative results (NA + PD);
N+: total number of positive results (PA+PD);
P(o): (PA+NA)/N; and
P(e): {(positive recovery in real-time PCR / total number of tested samples (N)) x (negative recovery in real-time PCR / total number of tested samples (N))} +{(negative recovery in LAMP / total number of tested samples (N)) x (negative recovery in real-time PCR / total number of tested samples (N))}.

Figure S1. Specificity of the RT-LAMP using an *in-house* developed POC device (PATHPOD, http://coronadx-project.eu/diagnostic-kits/pathpod/). 1A: *Escherichia coli*, 2A: *Streptococcus pyogenes*, 3A: Avian influenza viruses (AIV), 1B: *Klebsiella pneumonia*, 2B: *Pseudomonas auruginosa*, 1C: *Streptococcus pneumonia*, 2C: SARS-CoV control, 1D: *Staphylococcus aureus*, 2D: MERS-CoV control, 3D: Paramyxovirus, 4A: negative control and 4D: positive SARS-CoV-2 control. Please note that SARS-CoV-2 is amplified, whereas the previous SARS-CoV is not.

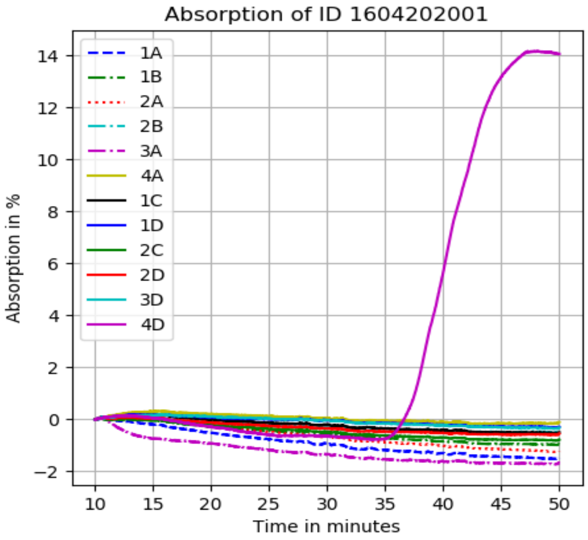

Figure S2. Sensitivity of rRT-LAMP assay which containing only dTTP (no dUTP) (A) using fluorescence detection method and (B) using turbidity detection method. In both A and B, a serial 5-fold dilution of a positive clinical sample was prepared.1: 5 times dilution, 2: 25 times dilution, 3: 125 times dilution, 4: 625 times dilution, 5: 3.125 times dilution, 6: 15.625 times dilution, 7: 78.125 times dilution, NC: negative control.

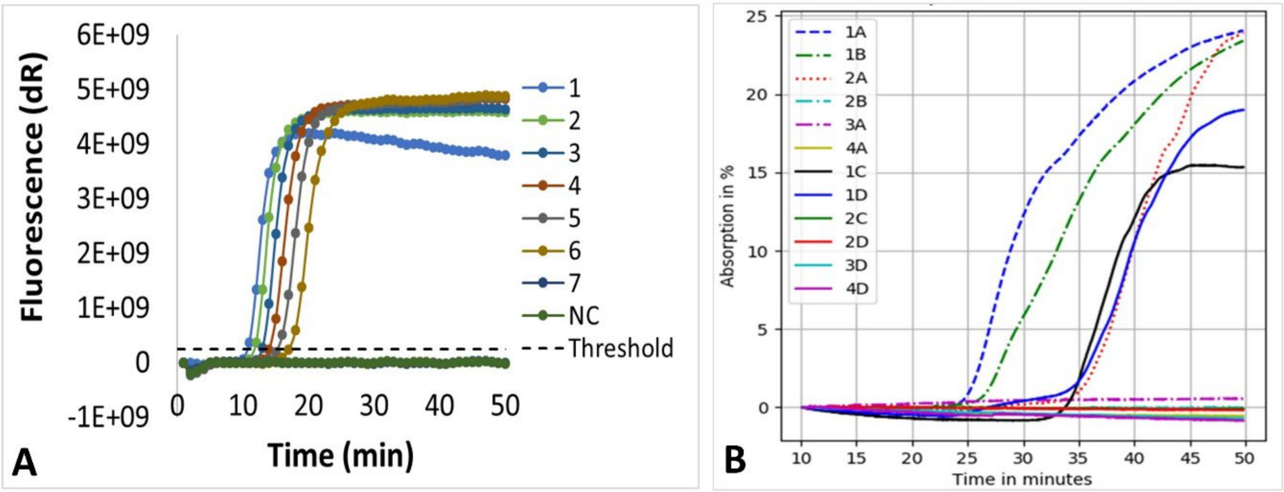

Table S1: T_t values and C_t values of clinical samples used in this study

No. samples	Cod-UNG RT-rtLAMP (T_t)	rRT-PCR (C_t)
1	No T_t	No C_t
2	No T_t	No C_t

3	No T_t	No C_t
4	No T_t	No C_t
5	No T_t	No C_t
6	No T_t	No C_t
7	No T_t	No C_t
8	No T_t	No C_t
9	No T_t	No C_t
10	No T_t	No C_t
11	No T_t	No C_t
12	No T_t	No C_t
13	No T_t	No C_t
14	No T_t	No C_t
15	No T_t	No C_t
16	No T_t	No C_t
17	No T_t	No C_t
18	No T_t	No C_t
19	No T_t	No C_t
20	No T_t	No C_t
21	10.27	8.99
22	15.65	23.52
23	No T_t	24.44
24	13.19	13.56
25	11.16	10.33
26	10.24	9.47
27	13.39	13.52
28	18.68	23.96
29	12.38	12.07
30	13.98	14.97
31	12.89	13.16
32	13.76	15.05
33	15.48	17.3
34	14.65	19.9
35	15.14	17.81
36	13.8	15.45
37	14.49	16.56
38	13.77	16.21
39	12.98	12.81
40	13.43	15.05
41	13.56	14.5
42	14.75	16.8
43	13.37	13.54
44	11.66	10.23
45	10.73	10.74
46	19.34	25.71
47	15.21	17.53

48	11.68	12.44
49	12.09	12.59
50	12.46	15.96
51	14.22	16.22
52	20.66	26.11
53	15.78	20.68
54	12.09	11.78
55	11.19	10.33
